# The calcium sensor CBL7 is required for *Serendipita indica*‐induced growth stimulation in *Arabidopsis thaliana*, controlling defense against the endophyte and K^+^ homoeostasis in the symbiosis

**DOI:** 10.1111/pce.14420

**Published:** 2022-08-29

**Authors:** Marta‐Marina Pérez‐Alonso, Carmen Guerrero‐Galán, Adrián González Ortega‐Villaizán, Paloma Ortiz‐García, Sandra S. Scholz, Patricio Ramos, Hitoshi Sakakibara, Takatoshi Kiba, Jutta Ludwig‐Müller, Anne Krapp, Ralf Oelmüller, Jesús Vicente‐Carbajosa, Stephan Pollmann

**Affiliations:** ^1^ Centro de Biotecnología y Genómica de Plantas, Universidad Politécnica de Madrid (UPM)–Instituto Nacional de Investigación y Tecnología Agraria y Alimentación (INIA/CSIC) Campus de Montegancedo Pozuelo de Alarcón (Madrid) Spain; ^2^ Umeå Plant Science Center Umeå University Umeå Sweden; ^3^ Department of Plant Physiology, Matthias Schleiden Institute of Genetics, Bioinformatics and Molecular Botany Friedrich‐Schiller‐University Jena Jena Germany; ^4^ Centro de Investigación de Estudios Avanzados del Maule Universidad Católica del Maule Talca Chile; ^5^ RIKEN Center for Sustainable Resource Science Tsurumi Yokohama Japan; ^6^ Department of Applied Biosciences, Graduate School of Bioagricultural Sciences Nagoya University Nagoya Japan; ^7^ Institute of Botany Technische Universität Dresden Dresden Germany; ^8^ Université Paris‐Saclay, INRAE, AgroParisTech Institut Jean‐Pierre Bourgin Versailles France; ^9^ Departamento de Biotecnología‐Biología Vegetal, Escuela Técnica Superior de Ingeniería Agronómica, Alimentaria y de Biosistemas Universidad Politécnica de Madrid (UPM) Madrid Spain

**Keywords:** Ca^2+^ signalling, endosymbiosis, growth promotion, plant defense, plant performance, plant−microbe interaction

## Abstract

Calcium is an important second messenger in plants. The activation of Ca^2+^ signalling cascades is critical in the activation of adaptive processes in response to environmental stimuli. Root colonization by the growth promoting endophyte *Serendipita indica* involves the increase of cytosolic Ca^2+^ levels in *Arabidopsis thaliana*. Here, we investigated transcriptional changes in Arabidopsis roots during symbiosis with *S. indica*. RNA‐seq profiling disclosed the induction of *Calcineurin B‐like 7* (*CBL7*) during early and later phases of the interaction. Consistently, reverse genetic evidence highlighted the functional relevance of CBL7 and tested the involvement of a CBL7‐CBL‐interacting protein kinase 13 signalling pathway. The loss‐of‐function of *CBL7* abolished the growth promoting effect and affected root colonization. The transcriptomics analysis of *cbl7* revealed the involvement of this Ca^2+^ sensor in activating plant defense responses. Furthermore, we report on the contribution of CBL7 to potassium transport in Arabidopsis. We analysed K^+^ contents in wild‐type and *cbl7* plants and observed a significant increase of K^+^ in roots of *cbl7* plants, while shoot tissues demonstrated K^+^ depletion. Taken together, our work associates CBL7 with an important role in the mutual interaction between Arabidopsis and *S. indica* and links CBL7 to K^+^ transport.

## 1 INTRODUCTION

Plants have evolved in spatial proximity to a multitude of different microorganisms in their surroundings. Their constant interaction with commensal, symbiotic and pathogenic microorganisms shaped highly specialized ecosystems in which plants find their niches and flourish. Since the initial description of the concept of mutual coexistence between dissimilar organisms (de Bary, 1879; Hertig et al., 1937), our knowledge regarding the symbiotic associations of plants with microorganisms has substantially advanced. Numerous studies unambiguously demonstrate that plant−microbe interactions are important to the structure, function and health of plant communities, and that symbiotic fungi contribute to the adaptations of plants to environmental stresses (Rodriguez et al., 2004).

*Serendipita indica* (formerly termed *Piriformospora indica*) is an axenically cultivable root colonizing plant endophyte of the order Sebacinales with an exceptionally broad host range (Mensah et al., 2020; Verma et al., 1998; Weiss et al., 2016). *S. indica* promotes plant performance and biomass production (Peskan‐Berghofer et al., 2004; Vadassery & Ranf, Drzewiecki, et al., 2009; Varma et al., 1999), enhances nutrient assimilation (Bakshi et al., 2017; Prasad et al., 2018), and confers increased biotic and abiotic stress tolerance to its host plants (Jogawat et al., 2016; Sun et al., 2014; Waller et al., 2005). The establishment of the symbiosis between *S. indica* and *Arabidopsis thaliana*, and the therewith coupled plant growth promotion, involves the perception of conserved microbial components by the plant, generally termed microbial‐associated molecular patterns (MAMPs), through specific pattern‐recognition receptor proteins. Pattern recognition, in turn, provokes a multitude of downstream events, including MAMP‐triggered immunity (Millet et al., 2010) and the induction of early plant defence responses, which comprise the deposition of callose and the production of defence‐related secondary metabolites, for example, phytoalexins, glucosinolates and camalexin (Jacobs et al., 2011; Lahrmann et al., 2015). At later stages of the infection, the controlled reduction of plant defence responses becomes paramount to facilitate the establishment of the mutual interaction between the endophyte and its host plant. In this respect, balancing of plant hormone contents and the tight control of indole glucosinolates are reported to play essential roles (Lahrmann et al., 2015; Nongbri et al., 2012; Xu et al., 2018).

The elevation of cytosolic Ca^2+^ concentrations in Arabidopsis root cells through the influx of Ca^2+^ via the Cyclic nucleotide gated channel 19 represents a further critical asset in consolidating the plant−fungus interaction (Jogawat et al., 2020; Vadassery & Ranf, Drzewiecki, et al., 2009). Calcium is an essential plant macronutrient that plays an important role in plant growth and development. At the same time, Ca^2+^ serves as an important second messenger in plants that is involved in orchestrating adequate responses to external signals, including biotic stresses (Thor, 2019). Cytosolic Ca^2+^ concentrations show highly dynamic and specific spatiotemporal patterns, which are governed by the type and intensity of the perceived stimulus (Pivato & Ballottari, 2021). Depending on the particular stimulus, plant cells respond by producing specific Ca^2+^ signatures that differ in their frequency, amplitude and duration (Batistič & Kudla, 2012). To decipher the different Ca^2+^ signatures, plants possess a broad set of different sensor molecules that either directly modify target proteins through phosphorylation or act through their physical interaction with specific partner proteins, including protein kinases (Kudla et al., 2018). This diverse set of Ca^2+^ sensor molecules encompasses Calmodulins (CaMs), Calmodulin‐like proteins (CMLs), Ca^2+^‐dependent protein kinases (CDPKs), Calcineurin B‐like proteins (CBLs), as well as their interacting kinases (CIPKs). The latter forming a two‐component system in which the CBLs act as Ca^2+^ sensors that relay their stimulation to specific CIPKs, which subsequently interact with downstream target proteins, such as nutrient transporters or ion channels (Liu & Tsay, 2003; Maierhofer et al., 2014; Ragel et al., 2015). In particular, the regulation of the high affinity potassium transporter HAK5 and the highly selective potassium channel AKT1 by CIPK23 is well documented (Lan et al., 2011; Lee et al., 2007; Ragel et al., 2015). However, there is also evidence for an interaction of CIPKs with diverse components of the abscisic acid response pathway, including the corepressors ABI1 and ABI2 (Ohta et al., 2003). The Arabidopsis genome contains 10 *CBL* and 26 *CIPK* genes. Among each other, the CBLs and CIPKs form functional complexes and it is noteworthy that CBLs are not promiscuously interacting with all CIPKs, but show preferences and interact only with a specific subset of CIPKs to facilitate efficient signal transduction and integration (Kudla et al., 2010). Overall, the specific combination of CBL‐CIPK modules is the key to provide versatility and flexibility in the regulation of a multitude of external stimuli that marshal ion transport in plants (Tong et al., 2021). In addition to the regulation of K^+^ transporters, CBL‐CIPK interactions have recently been described to control the polarization of plasma membranes (Yang et al., 2019). In this manner, CBL‐CIPKs can also indirectly regulate voltage‐gated K^+^ channels, such as the outward‐rectifying K^+^ channels SKOR and GORK (Dreyer et al., 2004).

Up to date, the functional role of Calcineurin B‐like 7 (CBL7) is only partially elucidated. A recent study associates CBL7 with the regulation of plant responses towards low nitrate in Arabidopsis (Ma et al., 2015). The work highlights a substantial expression of *CBL7* in root tissues and its induction under nitrogen and nitrate limiting conditions. Moreover, the authors report on the involvement of CBL7 in the transcriptional regulation of two high‐affinity nitrate transporter genes, *NRT2.4* and *NRT2.5*, without providing evidence for a molecular mechanism that could explain how the downstream genes are targeted. It is speculated that the localization of CBL7 to the nucleus facilitates its interaction with nitrate‐starvation response‐related transcription factors (TFs), such as NLP7 (Kiba & Krapp, 2016; Konishi & Yanagisawa, 2013; Krouk & Kiba, 2020; Marchive et al., 2013).

In this study, we identified the Ca^2+^ sensor CBL7 as an essential molecular component in the interaction between the root colonizing endophyte *S. indica* and *A. thaliana*. *CBL7* is consistently induced upon root infection with the fungus, not only at early stages of the infection, but also at later phases. Furthermore, we were able to demonstrate that the availability of CBL7 is crucial for the development of the fungus‐mediated plant growth promotion and for the proper distribution of potassium in the plant. The comprehensive transcriptomics analysis of the *cbl7* mutant grown with and without the fungus in comparison to corresponding wild‐type plants additionally pinpointed a role of CBL7 in harmonizing plant defense responses for the long‐term interaction between the endophyte and Arabidopsis. Taken together, our results establish CBL7 as a novel key component required for the successful establishment of the symbiosis between *S. indica* and *A. thaliana*.

## 2 MATERIALS AND METHODS

### 2.1 Biological material and growth conditions

In this work, we used the *A. thaliana* Col‐0 (N1092) and Col‐3 (N28171) backgrounds as references. We obtained the mutant alleles CBL‐interacting protein kinase 13 (*cipk13‐1)* (SALK_124748C) and *cipk13‐2* (SALK_130671), as well as *cbl7‐1* (SAIL_201_A01) from the Nottingham Arabidopsis Stock Centre (NASC). T‐DNA insertion lines were genotyped as previously described (Alonso et al., 2003), using the primer pairs listed in Supporting Information: Data Sheet 1. The T‐DNA insertion mutants *cbl7‐2* and *hak5* have previously been described (Ma et al., 2015; Ragel et al., 2015). Plants were grown on Petri dishes containing solidified ½ MS medium supplemented with 1% sucrose (w/v) (Murashige & Skoog, 1962). Plant growth proceeded in a growth chamber under strictly controlled environmental conditions (16 h light, 8 h darkness, constant temperature of 22°C, 100−105 µmol photons m^−2^ s^−1^ photosynthetically active radiation). In addition, we used *S. indica* strain DSM 11827, which was obtained from the German Collection of Microorganisms and Cell Cultures (DSZM) in Braunschweig, Germany, and the plant growth‐promoting fungus *Penicillium minioluteum* that has been extracted from roots of *Chenopodium quinoa* grown in the Atacama Desert (González‐Teuber et al., 2018). The fungi were grown at 28°C in darkness on solidified arginine phosphate (AP) medium (Rodríguez‐Navarro & Ramos, 1984) and refreshed weekly.

### 2.2 Root growth promotion assay

Surface‐sterilized Arabidopsis seeds were plated on vertical ½ MS plates. After stratification (2 days at 4°C), the plates were transferred to the growth chamber and the seedlings were grown vertically for 1 week. Thereafter, four to five seedlings were transferred to Petri dishes containing solidified Plant Nutrition Medium (PNM) supplemented with 50 mM NaCl (Johnson et al., 2013). Each seedling was then associated with a 5 mm Ø medium plug extracted from either sterile AP plates (control) or from AP plates harbouring a 1‐week‐old *S. indica* mycelium (cocultivation). The PMN plates with the control seedlings and the seedlings cocultivated with the fungus were further kept in a growth chamber maintained at 23.5°C, 16/8 h photoperiod, 100 µmol photons m^−2^ s^−1^ light intensity for another 10 days. After that time, the plants were photographed for the further analysis of the root system and the plant material was either used for RNA extraction or the determination of the fresh weight.

### 2.3 Quantitative analysis of root system architecture traits

The stimulation of root growth is a well‐described trait in the interaction of *S. indica* with its host plant. With the aim to quantify the effect of *S. indica* in the different genotypes and treatments, respectively, photographs of the plates were captured with a digital camera at a fixed distance of 29 cm. Using Adobe Photoshop CC, the images were cropped to a height of 14 cm maintaining only the part containing the root system and converted to black and white images. The root network traits of the plants in the prepared images were then analysed using the GiA Roots software (Galkovskyi et al., 2012). Further processing of the images included their segmentation employing global thresholding (Binary_inverted) and Gaussian adaptive thresholding. For the comparative analysis of alterations of the root system architecture, the total network area and total network length was used as readout. Taking the biological variability of the system into account, 24 individual plants per genotype and growth condition were analysed, respectively.

### 2.4 Total RNA extraction, library construction and RNA‐seq analysis

To study transcriptional alterations provoked by either the cocultivation of Arabidopsis roots with *S. indica* or the functional knockout of *CBL7*, total RNA from 100 mg plant roots was extracted as previously described (Oñate‐Sánchez & Vicente‐Carbajosa, 2008). The quality and concentration of the extracted RNA was tested by absorbance analysis using a Nanodrop® ND‐1000 spectrophotometer (Thermo Fisher Scientific). After an additional confirmation of the RNA sample integrity on a Bioanalyzer 2100 (Agilent) by the Novogene Genomics Service, the service laboratory proceeded with the library construction and RNA sequencing (PE150) on Illumina NovaSeq™ 6000 platforms. The Novogene Genomics Service also provided basic data analysis applying their RNAseq pipeline. This included data filtering and sequence alignment using HISAT2 v2.0.5 with default parameters (Kim et al., 2019), transcript quantification with HTSeq v0.6.1 with ‐m union parameter (Anders et al., 2014) and differential gene expression analysis employing the DESeq. 2 v1.22.2 algorithm with a cut‐off value of an adjusted *p*< 0.05 (Love et al., 2014). For each genotype and treatment, respectively, three biological replicates were processed. The resulting *p* values were adjusted for multiple testing using the Benjamini−Hochberg correction (Benjamini & Hochberg, 1995). Along with the adjusted *p* value [false discovery rate (FDR)] of <0.05 an absolute differential expression of log_2_ fold change (FC) ≥1.25 was chosen to select differentially expressed genes (DEGs). The functional classification of DEGs was performed using the MapMan v3.6 software (Thimm et al., 2004), paying special attention to DEGs related with plant defense and nutrient assimilation. Furthermore, functional relationships between the DEGs were investigated using the applications stringApp v1.7 (Doncheva et al., 2019), MCODE v2.0 (Bader & Hogue, 2003), EnrichmentMaps v3.3.3 (Merico et al., 2010) and ClueGO v2.5.8 (Bindea et al., 2009) in Cytoscape v3.9.0 (Shannon et al., 2003).

### 2.5 qPCR analysis

Real‐time quantitative RT‐PCR was conducted as previously described (Pérez‐Alonso et al., 2021). In brief, total RNA from three different biological samples was converted into cDNA using M‐MLV reverse transcriptase and oligo(dT)_15_ primer. Two nanograms of cDNA was then used as template for the qPCR reactions, which were conducted in triplicate (technical replicates). The oligonucleotide pairs used in the experiments are given in Supporting Information: Data Sheet 1. The reactions were monitored on a Lightcycler 480 Real‐time PCR system (Roche Diagnostics). Differential gene expression in Arabidopsis was analysed by using the comparative $2^{‐\Delta \Delta C_{t}}$method (Livak & Schmittgen, 2001) with *Adenine phosphoribosyl transferase 1* (*APT1*, At1g27450) as reference gene (Jost et al., 2007). Root colonization with *S. indica* was monitored with a primer pair for fungal translation elongation factor EF‐1α (*TEF1*) (Bütehorn et al., 2000). The fungal *TEF1* cDNA levels were expressed relative to the plant *Glycerinaldehyde‐3‐phosphate dehydrogenase C2* (*GAPC2*, At1g13440) cDNA levels. To exclude that the amplified DNA fragments stem from DNA of dead fungal tissues within the roots, all data presented here derived from cDNA libraries generated from RNA of colonized roots.

### 2.6 Trypan blue staining of fungal hyphae and spores

To visually inspect root colonization, 10−12 small root samples from control and cocolonized plants were employed. First, the root samples were thoroughly washed with deionized water. Next, the root samples were cut in 1 cm long pieces and incubated overnight in 10 N KOH. The root samples were then rinsed five times with sterile H_2_O, before they were incubated for 5 min in 0.1 N HCl. Finally, the samples were incubated in a 0.05% trypan blue solution (w/v), before they were partially decolorized with lactophenol over 10 min. Before the specimen were mounted on glass slides and examined by microscopy, they were washed once with 100% ethanol and three times with sterile H_2_O and stored in 60% glycerol (v/v).

### 2.7 K^+^ quantification

The analysis of endogenous cation contents of infected and control plants was performed according to Conchillo et al. (2021) in fractions of root and shoot samples. In brief, the fungi were first K^+^ depleted buy inoculating 100 ml AP medium containing 1 mM K^+^ with 1.5 × 10^3^ of the corresponding spores and incubating it for 1 week at 28°C. Next, the mycelia were harvested by centrifugation and transferred to fresh AP medium without K^+^. After another week of incubation, the K^+^ starved spores were harvested. At the same time, Arabidopsis seedlings were germinated on ½ MS plates for 1 week. Thereafter, the seedlings were transferred to square Petri dishes containing modified Hoagland agar medium adjusted to pH5.7 (Álvarez‐Aragón et al., 2016) without glucose and either no KCl or 1 mM KCl. After 1 week, the roots of 20 Arabidopsis seedlings were inoculated with 250 µl of a solution containing 1.5 × 10^6^ spores ml^−1^. Noninfected plants were grown under similar conditions. K^+^ quantification was performed after 1 week of cocolonization with the fungi. To avoid carry overs from the medium, roots were thoroughly rinsed with 10 mM MES‐Ca^2+^ pH 6.5. Next, root and shoot samples were dried, weight and extracted with 1 M HNO_3_ at ambient temperature over 36 h. The K^+^ contents of the supernatants were then determined by atomic emission spectroscopy. The results are given as the means and their standard errors of three independent experiments.

### 2.8 Statistical analysis

For statistical data assessment and the generation of box plots, JASP v0.16 was employed. The box plots show the median, quartiles and extremes of the compared experimental values. One‐way anova followed by Tukey's post hoc test or Student's *t*‐test were performed to statistically analyse the data. Sample sizes (*n*) for each experiment are given in the respective figure legends. Hierarchical clustering and heatmaps of selected gene expression levels across the different experiments was conducted using the Instant Clue software v0.10.10 (Nolte et al., 2018).

## 3 RESULTS

### 3.1 *CBL7* is induced in the symbioses between Arabidopsis and *S. indica*

**Figure 1:**
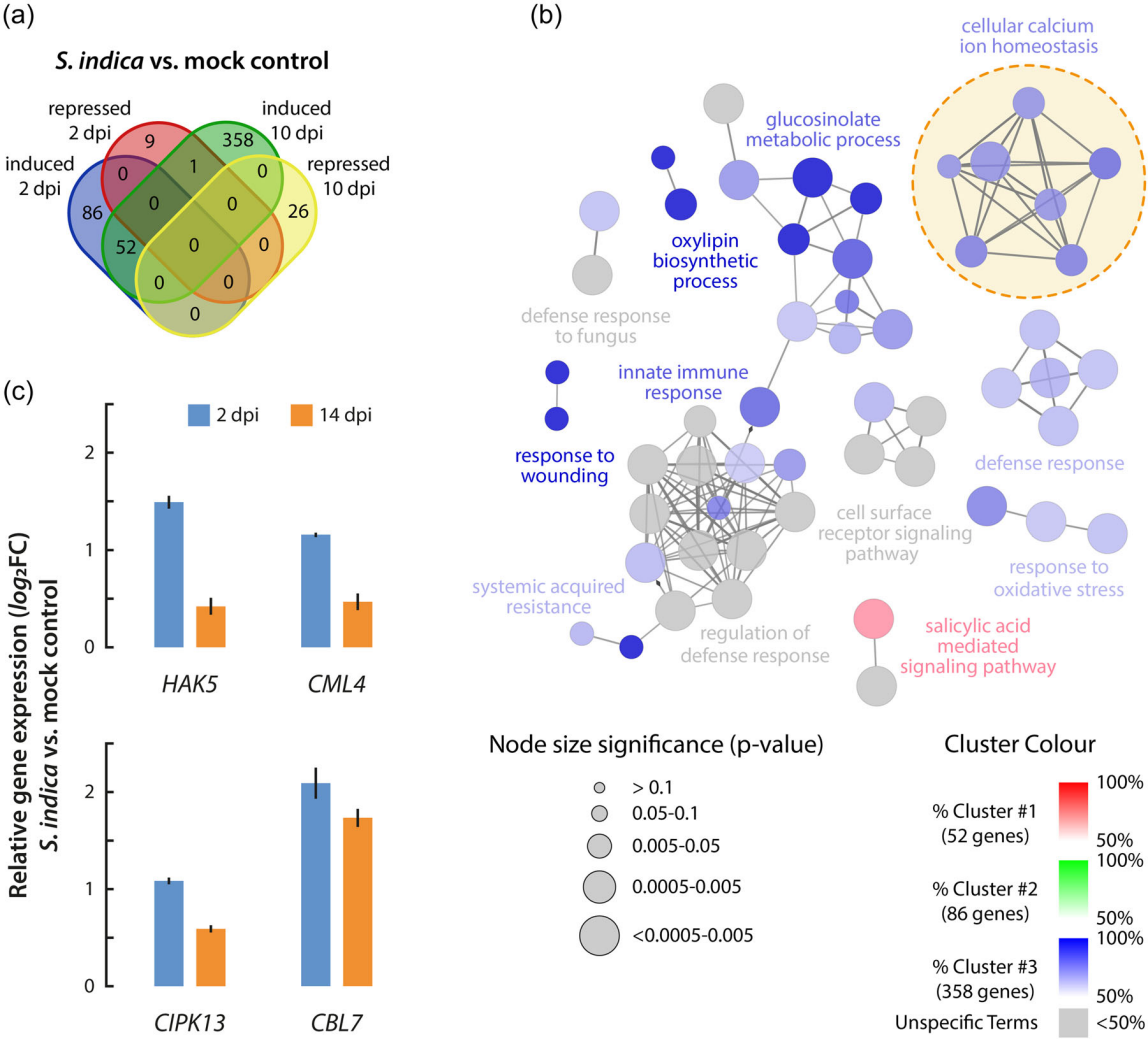
Transcriptional analysis of Arabidopsis seedlings cocultivated with *Serendipita indica*. (a) Venn diagram showing the numbers of differentially expressed genes in Arabidopsis plants 2‐ and 10‐days post infection with *S. indica* compared to control plants that were mock infected. (b) ClueGo analysis of induced DEGs. The figure shows the representative molecular function interaction among the targets. (c) qPCR analysis of transcriptional responses of identified target genes in plants that were cocultivated for 2 and 10 days, respectively, with *S. indica* compared to mock treated plants. The data represent means ± SE (*n* = 3). DEGs, differentially expressed genes; dpi, days post infection. [Color figure can be viewed at wileyonlinelibrary.com]

The growth promoting effect of *S. indica* on multiple host plant species has already been well‐characterized (Mensah et al., 2020). However, the precise molecular mechanism by which the fungal root endophyte induces plant growth remains largely elusive. To shed further light on the molecular basis of the interaction between *S. indica* and Arabidopsis, we took a comprehensive transcriptomics approach analysing the transcriptional differences between control plants and plants challenged with *S. indica* at both early [2 days post infection (dpi)] and later (10 dpi) stages of cocultivation. At the indicated time points, total RNA was isolated and, after library construction, subjected to mRNA‐seq analysis. The application of a Benjamini‐Hochberg false discovery rate (FDR) (*p*_adj._ ≤ 0.05) and setting the FC cut‐off to log_2_FC ≥ 1.25, identified 138 induced and 10 repressed genes, respectively, at 2 dpi, while we identified 411 induced and 26 repressed genes after 10 days of cocultivation (Supporting Information:Data Sheet 2). The expression data revealed that 9.8% of the DEGs were induced both after 2 and 10 days of cocultivation on PMN medium, while 16.2% and 67.3% of the DEGs were only induced in the early and later phases of the interaction, respectively (Figure 1a). Notably, only 6.8% of the DEGs were repressed under the tested conditions, and only one gene, *Senescence‐associated gene 13* (*SAG13*), turned out to be repressed during the establishment of the plant−fungus interaction, while being induced at later stages of the symbiosis. To gain further insight into the affected biological processes triggered by the fungus during the studied interaction, we performed gene ontology (GO) analyses of the induced DEG groups (Supporting Information:Image 1A). The analysis revealed a substantial enrichment of genes associated with stress and defense‐related GO terms. To decipher possible molecular mechanisms, we further employed a functionally enriched network analysis to identify biological interpretations and interrelations of functional groups in biological networks (Figure 1b) (Bindea et al., 2009). The network analysis largely confirmed the obtained enriched GO term classifications. In addition, it provided evidence for the enrichment of genes related to a group of GO terms associated with cellular Ca^2+^ homoeostasis and Ca^2+^ signalling processes, which attracted our attention and on which we followed up in this study.

To gain a better understanding of the role of Ca^2+^‐related processes in the mutual interaction between *S. indica* and Arabidopsis, we extracted the relative expression profiles of 88 Ca^2+^ signalling‐related genes from the nonfiltered RNA‐seq data sets and performed a hierarchical cluster analysis (Supporting Information: Image 1B). We have been particularly intrigued by the expression profile of the genes in Cluster 1, which contained the four genes *High affinity K* ^*+*^ *transporter 5* (*HAK5*), *Calmodulin‐like 4* (*CML4*), *CIPK13* and *CBL7*. The genes were consistently induced both at early and later stages of the plant−fungus interaction, which could be confirmed by qRT‐PCR analysis (Figure 1c). Activation of these genes might therefore be required both for the establishment and preservation of the mutual plant−fungus interaction.

### 3.2 CBL7 is required for fungus‐mediated growth promotion

**Figure 2:**
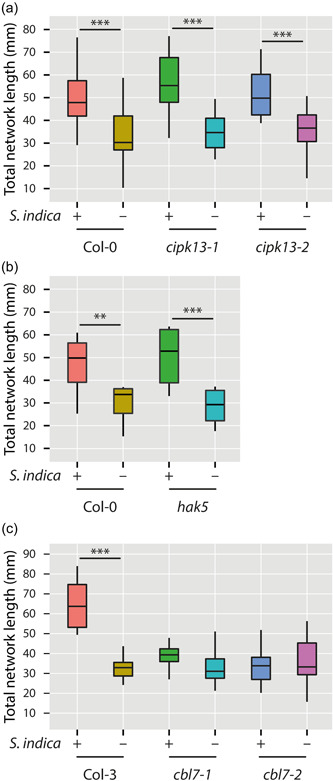
Total root network length of Arabidopsis wild‐type and mutant plants after 14 days of cocultivation with *Serendipita indica* or mock treatment. (a) Comparison of two *cipk13* T‐DNA insertion mutant alleles with Col‐0. (b) The growth promoting effect of *S. indica* is not affected in the *hak5* mutant. (c) Analysis of the fungus‐triggered growth promoting effect in Col‐3 and *cbl7* mutant plants. The box plots show the median, quartiles and extremes of the compared data sets (*n* = 24). Asterisks indicate significant differences between S. indica and mock treated samples. Student's *t*‐test: **p ≤ 0.01, ***p ≤ 0.001. *cbl7, Calcineurin B‐like; cipk13*, CBL‐interacting protein kinase 13. [Color figure can be viewed at wileyonlinelibrary.com]

CBL proteins are plant‐specific Ca^2+^ sensors that are predicted to decode calcium transients through interaction with CIPKs (Tang et al., 2020). It has previously been shown that several CBL proteins, that is, CBL1, CBL8, CBL9 and CBL10, interact with CIPK23 to regulate HAK5 transport activity (Ragel et al., 2015). Hence, we speculated that CBL7 and CIPK13 may physically interact with each other to control HAK5 activity as their downstream target, because of their shared expression profiles in response to the infection of Arabidopsis seedlings with *S. indica*. To test this hypothesis, we took a reverse genetics approach to assess the role of CBL7, CIPK13 and HAK5 in the fungus‐mediated promotion of plant growth. To do so, we used two independent T‐DNA insertion mutants for both *CBL7* and *CIPK13*, as well as the previously described *hak5* mutant. The mutants were grown for 7 days on ½ MS medium alongside with corresponding wild‐type control plants, before the seedlings were transferred to PNM medium, where they were either cocultivated with *S. indica* or a mock control. After 10 days, the vertically grown plants were photographed (Supporting Information: Figure S2), and the root system architecture was analysed using the GiA Roots software. The statistical assessment of the monitored parameters facilitated a quantitative comparison of the growth promoting effect elicited by *S. indica* in the different genotypes. As shown in Figure 2a, the functional knockout of *CIPK13* had no impact on the total root network length. Both *cipk13* alleles exhibited a significant growth promotion through the cocultivation with *S. indica*, similar to the response of the wild‐type control plants. The same observation was made for the *hak5* mutant (Figure 2b). In contrast, the loss‐of‐function mutants of *CBL7*, *cbl7‐1* and *cbl7‐2*, are characterized by a substantial reduction of the growth promoting effect observed in Col‐3 control plants (Figure 2c). The additional analysis of the total network area and biomass of the different groups of seedlings confirmed the obtained results (Table 1). Taken together, the results suggest that CBL7 plays an important role in the establishment/maintenance of the symbiosis, because on the one hand the functional inactivation of CBL7 coincides with a loss of the growth promoting effect normally exerted by the fungus. CIPK13 and HAK5, on the other hand, are seemingly likely not vital or replaceable in the plant−fungus interaction, given that the knockout mutants showed no significant alteration of their growth behaviour when infected with *S. indica*.

**Table 1:** Growth promoting effects on Arabidopsis plants mediated by the endophytic fungus

Genotype	Network area (cm^2^)			Root fresh weight (mg)		
	+ *Serendipita indica*	– *S. indica*	∆ ± *S. indica*	+ *S. indica*	– *S. indica*	∆ ± *S. indica*
Col‐3	7.3 ± 0.3	4.0 ± 0.3	3.3 ± 0.4***	126 ± 15	85 ± 15	41 ± 6**
*cbl7‐1*	6.0 ± 0.3	5.0 ± 0.4	1.0 ± 0.5	84 ± 11	64 ± 7	20 ± 4
*cbl7‐2*	5.4 ± 0.3	4.4 ± 0.2	1.0 ± 0.3	97 ± 15	73 ± 9	25 ± 5
Col‐0	3.8 ± 0.2	2.6 ± 0.2	1.2 ± 0.3**	96 ± 16	61 ± 12	36 ± 5**
*cipk13‐1*	4.3 ± 0.3	3.0 ± 0.2	1.3 ± 0.3***	112 ± 8	70 ± 6	42 ± 4**
*cipk13‐2*	3.3 ± 0.1	2.2 ± 0.2	1.1 ± 0.2***	105 ± 9	73 ± 10	31 ± 5**
*hak‐5*	4.7 ± 0.3	2.5 ± 0.2	2.2 ± 0.4***	100 ± 10	70 ± 13	31 ± 5*

*Note*: Differences in total root network area and root fresh weight were used as molecular marker of the growth promoting effect of *S. indica* on its host plants. Data represent means ± SE (*n* = 24). Asterisks indicate significant differences between *S. indica* and mock treated samples. Student's *t*‐test: *p ≤ 0.05, **p ≤ 0.01, ***p ≤ 0.001.

Abbreviations: *cbl7, Calcineurin B‐like*; *cipk13*, CBL‐interacting protein kinase 13.

### 3.3 A loss of *CBL7* interferes with potassium distribution in Arabidopsis

**Figure 3:**
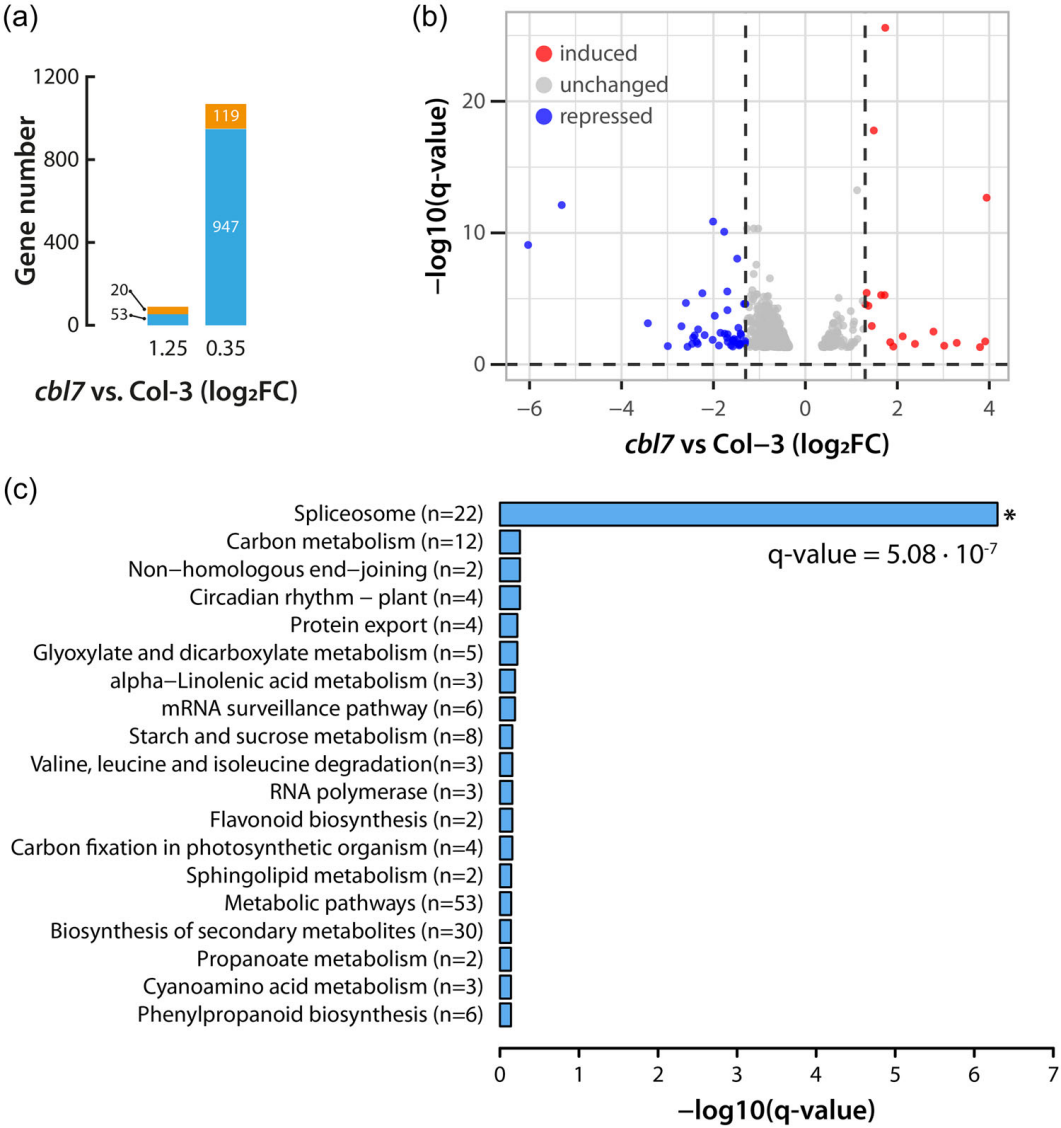
Analysis of transcriptional differences between *cbl7* mutant and wild‐type Col‐3 plants. (a) DEGs statistics for the *cbl7* mutant compared to Col‐3 control plants under control conditions. (b) Volcano plot of the distribution of all DEGs, mapping the 20 upregulated genes (red) and 53 down‐regulated genes (blue). The figure shows the relation between the significance [−log_10_ (*q* value)] and the strength (log_2_FC) of the differential expression. (c) Bar plot of the KEGG enrichment analysis of induced DEGs in *cbl7* compared to Col‐3 control plants under control conditions. Only the spliceosome KEGG term appeared to contain significantly enriched genes. Gene enrichment is given by ‐log_10_ (*q* value). *cbl7, Calcineurin B‐like;* DEGs, differentially expressed genes; FC, fold change. [Color figure can be viewed at wileyonlinelibrary.com]

**Figure 4:**
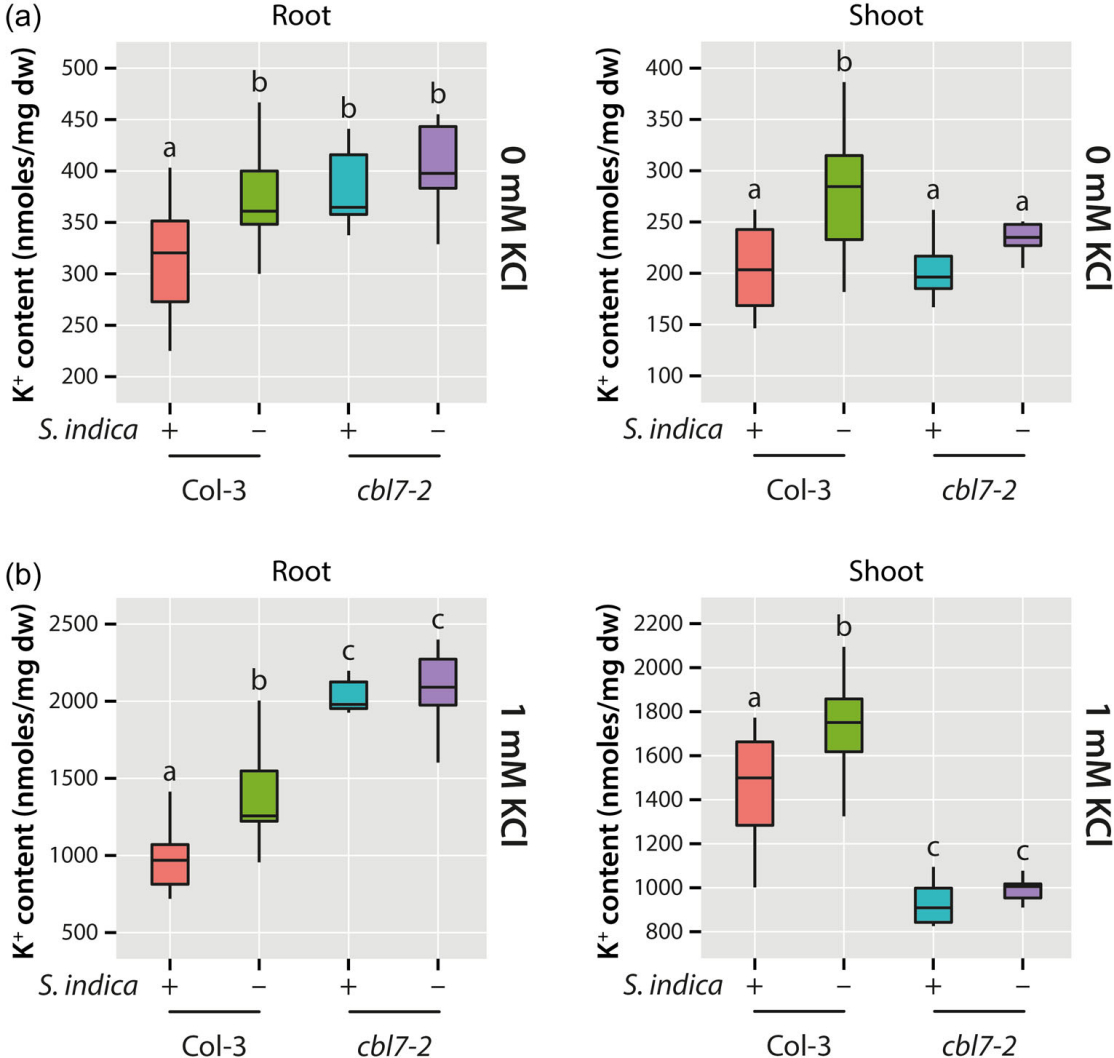
K^+^ contents of root and shoot tissues of *Serendipita indica* infected and noninfected control plants. The plants were grown in modified Hoagland medium under K^+^ starvation (0 mM KCl) (a) or at 1 mM KCl (b). The box plots show the median, quartiles and extremes of the compared data sets (*n*= 15). Different letters indicate significant differences between the compared conditions and genotypes analysed by ANOVA and a Tukey−Kramer post hoc test (*p* < 0.05). [Color figure can be viewed at wileyonlinelibrary.com]

To further investigate the biological functions of CBL7, we conducted another set of mRNA‐seq analyses comparing the transcriptional profiles of *cbl7‐2* and Col‐3 (wt) plants under control conditions. After applying the same threshold as before (FDR *p*_adj._ ≤ 0.05, log_2_FC ≥ 1.25), we identified only 20 induced and 53 repressed DEGs (Figure 3a,b), respectively. The GO analysis of this reduced number of DEGs did not provide evidence for the significant enrichment of any GO term. As the arbitrary chosen expression threshold provided no significant results, we repeated the analysis with less stringent threshold values (FDR *p*_adj._ ≤ 0.05, log_2_FC ≥ 0.35), giving 119 induced and 947 repressed DEGs, respectively (Figure 3a,b). Subsequent GO and KEGG enrichment analysis revealed the repression of 22 genes associated with the spliceosome (Figure 3c). Furthermore, we were able to identify the induction of 18 (*p*_adj._ = 3.34 × 10^−9^) and 12 (*p*_adj._ = 2 × 10^−3^) genes related to cell wall organization and ion transport GO terms, respectively (Supporting Information:Data Sheet 2). Among the latter genes, we found the high‐affinity nitrate transporter *NRT2.4* as well as the low‐affinity nitrate transporter *NPF2.9*. The involvement of CBL7 in the transcriptional regulation of nitrate transporters has previously been reported (Ma et al., 2015). Additionally, we again found that the high‐affinity potassium transporter gene *HAK5* was significantly induced (log_2_FC = 1.85, *p*_adj._ = 0.022). Although our previous results questioned the role of HAK5 in the plant−fungus interaction, the reiterated appearance of *HAK5* led us to analyse the potassium content in roots and shoots of *cbl7‐2* and Col‐3 to determine whether CBL7 could be involved in the regulation of potassium homoeostasis in Arabidopsis. For this, the K^+^ contents of fungus and mock‐treated mutant and wt seedlings were quantified by atomic emission spectroscopy. As shown in Figure 4, the potassium content of the *cbl7‐2* roots grown at 0 mM KCl was similar to that of the Col‐3 control plants. However, the previously described decrease in K^+^ contents in response to the inoculation with *S. indica* (Conchillo et al., 2021) was only observed in control plants, but not in *cbl7‐2*. Interestingly, the K^+^ level in the shoots of *cbl7‐2* plants was significantly lower than in the mock‐treated wild type, equal to the level observed for Col‐3 treated with the fungus. Although K^+^ levels showed a tendency to decrease in *cbl7‐2* shoots, there was no significant difference between mock and fungus‐treated *cbl7‐2* plants. Plants grown on plates containing 1 mM KCl showed a similar picture (Figure 4b), only wild‐type Col‐3 plants showed the expected decrease in K^+^ in response to the *S. indica* infection. Furthermore, we found a significant increase of K^+^ in *cbl7‐2* roots, while the K^+^ content in *cbl7‐2* shoots appeared to be significantly reduced. We have therefore concluded that CBL7 contributes to the regulation of the distribution of K^+^ in the plant. The functional loss of CBL7 is unequivocally linked to an altered K^+^ distribution profile, which is characterized by an increase of K^+^ in the roots, most likely due to a lack of transport of K^+^ to areal plant tissues. At the same time, our data suggest that CBL7 could also play a key role in the observed decrease in K^+^ levels in plants challenged with *S. indica*, as *cbl7* mutants present a reduced reduction of K^+^ when cocultivated with *S. indica*.

### 3.4 The *cbl7* mutant shows increased plant defense responses

**Figure 5:**
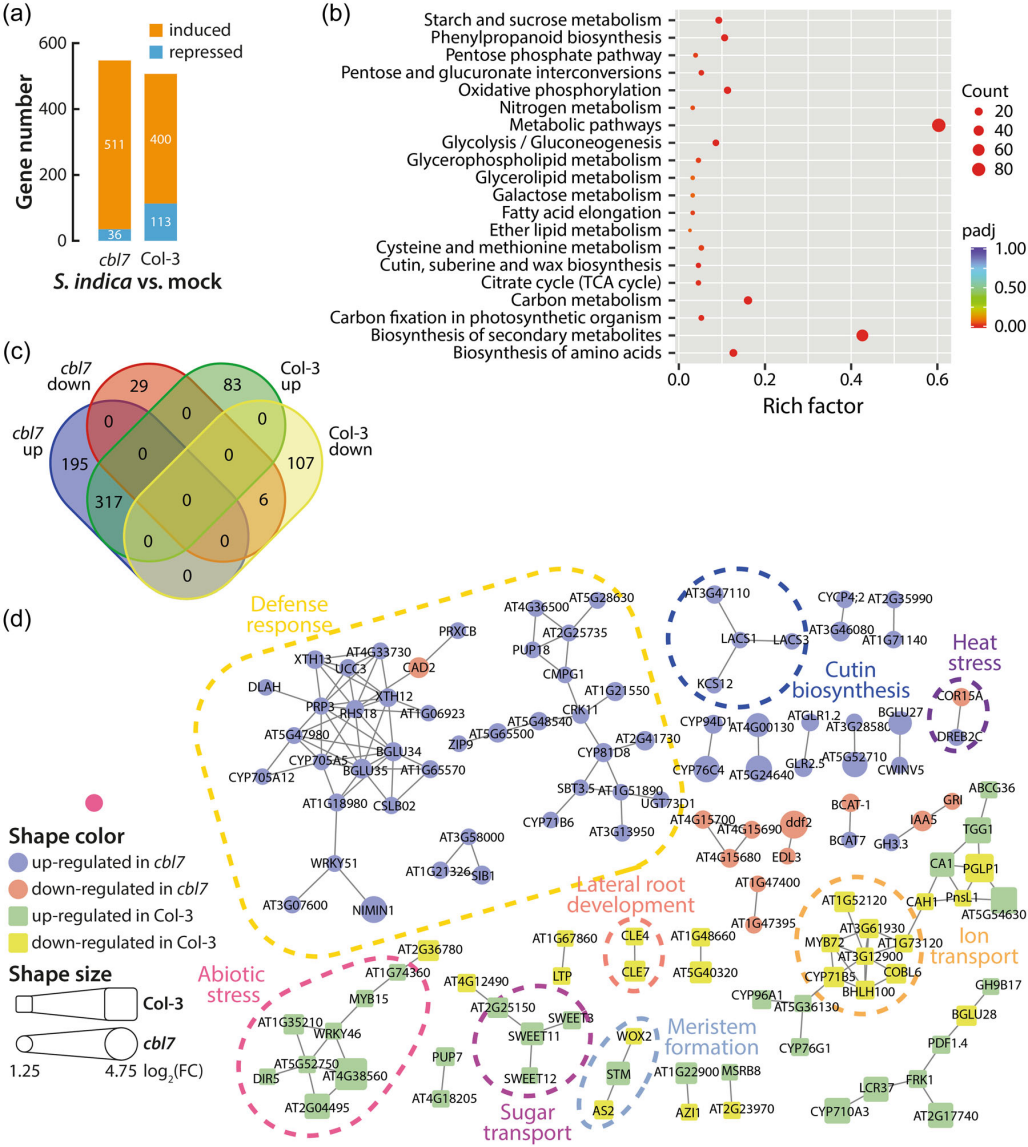
Transcriptomics analysis of mock and *Serendipita indica* treated the *cbl7* mutant plants to wild‐type Arabidopsis. (a) DEGs statistics for the *cbl7* mutant and Col‐3 control plants after cocultivation with *S. indica* for 14 days. (b) KEGG enrichment analysis of induced DEGs in *cbl7* cocultivated with the fungus versus similarly treated Col‐3 plants. Each circle in the figure represents a distinct KEGG pathway, and the circle size indicates the number of genes enriched in the corresponding metabolic pathway. The significance of the observed gene enrichment is represented by a colour gradient referring to the adjusted *p* value (*p*adj). (c) Venn diagram showing the numbers of DEGs in Col‐3 and *cbl7* 14‐days post infection with *S. indica* compared to control plants that were mock infected. (d) Functional network analysis of DEGs in *cbl7* and Col‐3. Up‐ and down‐regulated genes are differentiated by colour and shape. The shape size refers to the different levels of differential expression according to the log_2_FC levels observed in the RNAseq analysis. Functional associations are highlighted in the figure. *cbl7, Calcineurin B‐like;* DEGs, differentially expressed genes; FC, fold change. [Color figure can be viewed at wileyonlinelibrary.com]

The above results highlighted the transcriptional regulation of *CBL7* in response to the infection of Arabidopsis roots with *S. indica* and its involvement in K^+^ partitioning in the plant. With the goal of further exploring the role of CBL7 in the establishment and maintenance of the symbiosis between Arabidopsis and *S. indica*, we compared the mRNA‐seq data surveyed from *cbl7‐2* knockout plants and corresponding Col‐3 control plants infected with *S. indica* and mock treated, respectively (Supporting Information: Data Sheet 2). Quantitative analysis of differential gene expression revealed a substantial increase in induced genes in the *cbl7* mutant relative to wt (Figure 5a). Furthermore, the examination of the possible logical relations between the two data sets revealed four nonoverlapping and two overlapping groups for DEGs in *cbl7‐2* and Col‐3 (*S. indica* vs. mock) (Figure 5c). Next, we performed a GO and functional network analysis to identify biological processes significantly affected by the loss of *CBL7*. The main alterations in the *cbl7* mutant could be associated with plant defense‐related processes, especially with metabolic pathways that are involved in the biosynthesis of secondary metabolites related to plant defense (Figure 5b,d). The enriched genes in these GO terms included a substantial number of cytochrome P450 enzymes, such as *CYP71B3*, *CYP71A12*, *CYP71A13* and *CYP71B15* (PAD3), which are known to participate in glucosinolate and camalexin biosynthesis (Frerigmann et al., 2016; Glawischnig, 2007). Furthermore, we found an induction of the myrosinase genes *BGLU34* and *BGLU35* that are involved in the turnover of glucosinolates (Wittstock & Burow, 2010). The observation of an induction of plant defense‐related compounds is further supported by the induction of TFs *NAC042*, *WRK33* and *WRK51*, which have been linked with the regulation of camalexin and indole glucosinolate biosynthesis (Birkenbihl et al., 2012; Frerigmann & Gigolashvili, 2014; Saga et al., 2012; Zhou et al., 2020). Furthermore, we also observed an induction of *WRKY70*, a TF involved in modulating cell wall‐related defense responses (Li et al., 2017). Moreover, the functional analysis of the transcriptomics data revealed an enrichment of glutathione S‐transferases among the induced genes in *cbl7‐2*, which included the genes *GSTU10*, *GSTU12*, *GSTF3*, *GSTF6* and *GSTF7*. Glutathione S‐transferases are readily induced by a wide range of stress conditions, including bacterial and fungal infections (Dixon et al., 2002; Gullner et al., 2018). Considering that all these processes are observed in the *cbl7* loss‐of‐function mutant, it must be concluded that CBL7 is involved in the suppression of multilayered defense responses to facilitate the establishment of *S. indica* in the root apoplast. However, other processes that appear enriched in *S. indica* challenged wt plants but not in *cbl7*, such as the induction of sucrose transporter genes and *WRKY46* orchestrated abiotic stress responses (Chen et al., 2010, 2012; Ding et al., 2015), or the repression of *bHLH100* and *MYB72* mediated metal ion homoeostasis (Sivitz et al., 2012; Zamioudis et al., 2015), are likely to contribute to the significant difference in growth promotion between *cbl7* and wt triggered by the fungus.

**Figure 6:**
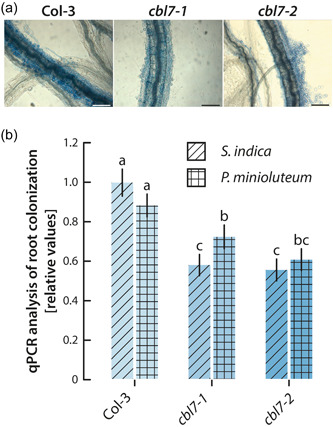
Analysis of the colonization of Col‐3 and *cbl7* roots with *Serendipita indica*. (a) Trypan blue stain of root segments at 1−2 cm distance from the root tip. The figure shows representative pictures for the three studied genotypes after 14 days of cocultivation with *S. indica*. (b) Quantitative assessment of root colonization by *S. indica* and *Penicillium minioluteum* using qPCR analysis of the corresponding *TEF1* genes of the two fungi. Depicted are means ± SE of three independent biological experiments performed in triplicate. The Arabidopsis *GAPC2* gene served as endogenous control. Different letters indicate significant differences between the compared conditions and genotypes analysed by ANOVA and a Tukey−Kramer post hoc test (*p* < 0.05). *cbl7, Calcineurin B‐like; GAPC2, glycerinaldehyde‐3‐phosphate dehydrogenase C2*. [Color figure can be viewed at wileyonlinelibrary.com]

Based on our hypothesis of an increased defense response in the *cbl7* knockout mutant, it must be expected that the roots of mutant plants are less well colonized by plant growth promoting fungi than comparable wt roots. To prove this, we analysed root colonization in *cbl7* and Col‐3 by qPCR and trypan blue staining. Indeed, root colonization of the *cbl7* mutant was significantly reduced, both for *S. indica* and *P. minioluteum* (Figure 6). Our experiments have shown that CBL7 plays a critical role in maintaining the equilibrium between symbiotic interaction and plant defense that keeps the load of endophyte fungi under control.

## 4 DISCUSSION

Calcium signalling plays an important role in the regulation of a wide array of biological processes in eukaryotic physiology, including pathogenic and beneficial plant−microbe interactions (Vadassery & Oelmüller, 2009). In this study we showed that CBL7 is among the very few genes that display a consistent positive transcriptional response in Arabidopsis wild‐type plants infected with the beneficial root endophyte *S. indica*, both at early and later stages of the interaction (Figure 1). The coexpression of *CBL7*, *CIPK13* and *HAK5* prompted us to speculate that there might be a relevant connection between these components, which could regulate the uptake of potassium into host plants and, thus, their nutrition with this essential macronutrient. The activation of HAK5 through phosphorylation by CIPK23 has previously been described for Arabidopsis and tomato plants (Amo et al., 2021; Ragel et al., 2015). Hence, an interaction cascade between the three components appeared possible. However, when we analysed the relevance of the different components with respect to their impact on the growth promoting effect on Arabidopsis roots, we had to realize that only CBL7, but neither CIPK13 nor HAK5 interfered with growth promotion (Figure 2). While both *cbl7* mutant alleles showed a severe loss of growth promotion, none of the other investigated mutants demonstrated significant differences to wt. Although CIPKs and potassium transporters form large gene families, which opens the possibility that their loss might be compensated by other family members, it must be concluded that CIPK13 and HAK5 are not critical for the fungus‐triggered plant growth promotion. The possible plastid localization of CIPK13 further argues against an interaction with CBL7 in vivo (Schliebner et al., 2008).

Previous work on the role of CBL7 highlighted its interaction with the *A. thaliana* plasma membrane proton ATPase 2 (AHA2) (Yang et al., 2019). In their model, the CBL7/AHA2 complex is further stabilized by the interaction with protein kinase SOS2‐like 5 (PKS5), also referred to as CIPK11. The authors suggest that the Ca^2+^‐mediated dissociation of the CBL7/CIPK11/AHA2 complex under saline‐alkali stress conditions translates into the activation of AHA2. Activation of AHA2, in turn, leads to hyperpolarization of the plasma membrane, which is likely to affect the transport activity of *Shaker*‐like K^+^ channels in the root. SKOR, an outward‐rectifying K^+^ channel, is reported to be crucial for the loading of K^+^ into the xylem and, thus, the long‐distant transport of K^+^ within the plant (Gaymard et al., 1998). Moreover, SKOR is known to form heteromeric outward‐rectifying K^+^ channel units with a second *Shaker*‐like channel, GORK (Dreyer et al., 2004). SKOR and GORK facilitate K^+^ transport only when the plasma membrane is depolarized (Dreyer & Blatt, 2009). A recent publication further pinpointed the importance of the complex interplay between different nutrient transporter systems and proton pumps in nutrient cycling in plants (Dreyer, 2021). Based on these observations, it must be assumed that a loss of CBL7 will likely result in a disrupted regulation of AHA2 activity and, consequently, a hyperpolarization of the plasma membrane, which entails a reduced transport activity of SKOR and GORK. As a result, the assimilated K^+^ would accumulate in the roots, and only reduced amounts of K^+^ would reach the shoot. In fact, our analysis of K^+^ levels in wild‐type and *cbl7* mutants corroborates this hypothesis (Figure 3). The *cbl7* mutant plants showed a pronounced increase of K^+^ contents in the roots, which we attribute to possible impairment of K^+^ xylem transport. Potassium depletion in the shoot could trigger the induction of *HAK5* in *cbl7*, as multiple independent studies demonstrated that *HAK5* is induced under K^+^ starvation (Ahn et al., 2004; Armengaud et al., 2004; Gierth et al., 2005; Shin & Schachtman, 2004). A recent study demonstrated the induction of *HAK5* in the host plant as a general consequence of the symbiotic interaction (Conchillo et al., 2021), which would also explain why we found *HAK5* under the consistently induced genes in wild‐type plants (Figure 1). Furthermore, the latter study demonstrated that the inoculation of Arabidopsis with *S. indica* does not improve the K^+^ nutrition of the host plant under K^+^ limiting conditions. Intriguingly, the authors showed that root colonization is stimulated under K^+^ limiting conditions, leading to the assumption that the endophyte may benefit from the relatively high cellular K^+^ concentrations in plant cells or apoplast. The K^+^ content in plant tissue was reported to be significantly reduced when plants were inoculated with *S. indica*. Our own work confirms this finding of reduced K^+^ levels in wt plants cocultivated with the fungus. On the contrary, the reduction of K^+^ levels upon infection with the fungus was largely absent in *cbl7* mutant plants, possibly because the flux of K^+^ from the host plant to the fungus is hampered by potassium channels/transporters. However, to validate this hypothesis further studies are needed. Nevertheless, together with the widely missing *S. indica*‐triggered growth promotion in inoculated *cbl7* mutants, this observation further underlined the vital role of CBL7 in the symbiotic plant−fungus interaction.

To further investigate the role of CBL7 in this context, we subjected the *cbl7‐2* mutant to additional RNA‐seq analyses. In a first analysis, we compared the transcriptional pattern of the *cbl7‐2* mutant with that of the corresponding wild type, Col‐3, under control conditions. After applying an arbitrary cut‐off value of log_2_FC ≥ 1.25, we identified only 73 DEGs that did not show enrichment of any biological process or function. Lowering the stringency of our analysis disclosed the repression of genes that are associated with the KEGG spliceosome term, including various splicing factors and the *suppressor‐of‐white‐apricot/surp domain‐containing protein* gene (Lorkovic et al., 2005). This suggests a possible impairment of alternative splicing processes in *cbl7*. It is, however, noteworthy that the gene that showed the strongest repression with a log_2_FC of –2.34, *MOS4‐associated complex subunit 5C* (*MAC5C*), does not appear to form part of the spliceosome associated MAC complex (Monaghan et al., 2010), but is more closely linked with processes related with secondary cell wall synthesis (Taylor‐Teeples et al., 2015). This possible relation of MAC5C with cell wall synthesis is consistent with the observed enrichment of cell wall biogenesis‐related genes, including the *hydroxyproline‐rich glycoprotein 1* (*HRGP1*) and the proline‐ and leucine‐rich extensin‐like family protein genes *EXT3*, *EXT4*, *EXT6*, *EXT10* and *LRX1*, respectively, which are moderately induced in *cbl7*. Extensin proteins play an important role in cell wall sensing. They are insoluble cell wall components that act as protein−protein interaction platforms to which peptide hormones and transmembrane receptors can bind, thereby relaying the perception of extracellular stimuli to the cytoplasm (Herger et al., 2019). These transcriptomic alterations possibly make the *cbl7* mutant more responsive to changes in its environment, including the perception of beneficial and pathogenic microbes on the root surface and in the apoplast.

This notion is further supported by the strongly induced plant defense response in *cbl7‐2*. When comparing the transcriptional responses of *cbl7‐2* plants infected with *S. indica* versus uninfected *cbl7‐2* control plants with the corresponding responses of Col‐3 plants, we found a significant enrichment of genes that fall into the secondary metabolite biosynthesis GO term in *cbl7‐2*. This group contains the TF genes *NAC042*, *WRK33* and *WRK51*, as well as the cytochrome P450 genes *CYP71B3*, *CYP71A12*, *CYP71A13* and *CYP71B15* (PAD3) encoding proteins involved in the biosynthesis of glucosinolates and camalexin, two secondary metabolites involved in the defense against pathogens (Glawischnig, 2007; Malka & Cheng, 2017). A previous study has already investigated the role of compounds derived from indole‐3‐acetaldoxime (IAOx) in the interaction of Arabidopsis with its root endophyte *S. indica* (Nongbri et al., 2012). The authors provide conclusive evidence that the infection of Arabidopsis with *S. indica* includes the induction of the formation of IAOx derived compounds during early stages of the interaction as a general defense response of the host plant. After the establishment of the symbiosis, the formation of indole glucosinolates and camalexin was reported to decrease. However, a certain wild‐type level of these compounds appears to be required to avoid excessive root colonization, as the lack of these compounds in the IAOx‐deficient *cyp79b2/cyp79b3* double mutant results in significantly increased root colonization, which is accompanied by a complete loss of fungus‐conferred plant growth promotion. Excessive root colonization is suggested to convert the beneficial interaction between *S. indica* and Arabidopsis into a physiological burden for the host plant. In case of the *cbl7‐2* mutant, the limitation of the formation of IAOx‐derived secondary metabolites is not working properly, because we still found these plant defense‐related genes induced after 14 days of cocultivation. Consequently, it must be concluded that the mutant overreacts to the biotic stress caused by the fungus, which results in a stronger defense response and a less efficient establishment of the symbiosis. Both the visual inspection of root colonization by Trypan blue staining and the quantification of root colonization by qPCR confirmed the reduced colonization of *cbl7* mutant roots with *S. indica* relative to those of wild‐type controls.

In summary, our work provides functional analyses of the cytoplasmic Ca^2+^ sensor CBL7 in the context of plant−fungus interactions. Based on the presented results, we propose that *CBL7* is induced during root infection of Arabidopsis with *S. indica* and contributes to the control of the classical plant defense. The loss of *CBL7* unequivocally blocks the normally observed promotion of *S. indica*‐triggered plant growth, likely through a reduced root colonization, as the long‐term harmony between the two symbionts is out of balance in the mutant. Furthermore, our work shed additional light on the so far undisclosed role of CBL7 in controlling potassium translocation in the plant body, most probably through its interaction with the plasma membrane ATPase AHA2. Under normal conditions, Ca^2+^ signals elicited by the root infection trigger the dissociation of the CBL7/CIPK11/AHA2 complex (Yang et al., 2019), which subsequently results in the hyperpolarization of the membrane. Consequently, this leads to the inactivation of outward rectifying potassium channels and a reduced flux of K^+^ into the xylem, which in turn results in an increase of potassium in the roots. In this context, it needs to be remarked that the expression of neither *SKOR* and *NPF7.3*/*NRT1.5* nor *SGN3*, which have previously been associated with K^+^ transport in Arabidopsis (Drechsler et al., 2015; Pfister et al., 2014), appeared to be significantly altered in the *cbl7‐2* mutant. It will be a thrilling future task to decipher whether impaired repression of plant defense responses or out‐of‐control potassium translocation in *cbl7* are the cause of the observed reduced root colonization and missing fungus‐triggered plant growth promotion.

## CONFLICT OF INTEREST

The authors declare no conflict of interest.

## Supporting information

Supporting information.

Supporting information.

Supporting information.

Supporting information.

Supporting information.

Supporting information.

## Data Availability

All data supporting the findings of this study are available within the paper and within its supplementary materials published online.

## References

[pce14420-bib-0001] Ahn, S.J. , Shin, R. & Schachtman, D.P. (2004) Expression of KT/KUP genes in Arabidopsis and the role of root hairs in K+ uptake. Plant Physiology, 134(3), 1135–1145. Available at 10.1104/pp.103.034660 14988478 PMC389937

[pce14420-bib-0002] Alonso, J.M. , Stepanova, A.N. , Leisse, T.J. , Kim, C.J. , Chen, H. , Shinn, P. et al. (2003) Genome‐wide insertional mutagenesis of *Arabidopsis thaliana* . Science, 301(5633), 653–657. Available at 10.1126/science.1086391 12893945

[pce14420-bib-0003] Álvarez‐Aragón, R. , Haro, R. , Benito, B. & Rodríguez‐Navarro, A. (2016) Salt intolerance in Arabidopsis: shoot and root sodium toxicity, and inhibition by sodium‐plus‐potassium overaccumulation. Planta, 243(1), 97–114. Available at 10.1007/s00425-015-2400-7 26345991

[pce14420-bib-0004] Amo, J. , Lara, A. , Martínez‐Martínez, A. , Martínez, V. , Rubio, F. & Nieves‐Cordones, M. (2021) The protein kinase SlCIPK23 boosts K^+^ and Na^+^ uptake in tomato plants. Plant, Cell and Environment, 44(12), 3589–3605. Available at 10.1111/pce.14189 34545584

[pce14420-bib-0005] Anders, S. , Pyl, P.T. & Huber, W. (2014) HTSeq—a python framework to work with high‐throughput sequencing data. Bioinformatics, 31(2), 166–169. Available at 10.1093/bioinformatics/btu638 25260700 PMC4287950

[pce14420-bib-0006] Armengaud, P. , Breitling, R. & Amtmann, A. (2004) The potassium‐dependent transcriptome of Arabidopsis reveals a prominent role of jasmonic acid in nutrient signaling. Plant Physiology, 136(1), 2556–2576. Available at 10.1104/pp.104.046482 15347784 PMC523322

[pce14420-bib-0007] Bader, G.D. & Hogue, C.W.V. (2003) An automated method for finding molecular complexes in large protein interaction networks. BMC Bioinformatics, 4(1), 2. Available at 10.1186/1471-2105-4-2 12525261 PMC149346

[pce14420-bib-0008] Bakshi, M. , Sherameti, I. , Meichsner, D. , Thürich, J. , Varma, A. , Johri, A.K. et al. (2017) *Piriformospora indica* reprograms gene expression in Arabidopsis phosphate metabolism mutants but does not compensate for phosphate limitation. Frontiers in Microbiology, 8, 1262. Available at 10.3389/fmicb.2017.01262 28747898 PMC5506084

[pce14420-bib-0009] Batistič, O. & Kudla, J. (2012) Analysis of calcium signaling pathways in plants. Biochimica et Biophysica Acta (BBA)—General Subjects, 1820(8), 1283–1293. Available at 10.1016/j.bbagen.2011.10.012 22061997

[pce14420-bib-0010] Benjamini, Y. & Hochberg, Y. (1995) Controlling the false discovery rate: a practical and powerful approach to multiple testing. Journal of the Royal Statistical Society: Series B (Methodological), 57(1), 289–300. Available at 10.1111/j.2517-6161.1995.tb02031.x

[pce14420-bib-0011] Bindea, G. , Mlecnik, B. , Hackl, H. , Charoentong, P. , Tosolini, M. , Kirilovsky, A. et al. (2009) ClueGO: a cytoscape plug‐in to decipher functionally grouped gene ontology and pathway annotation networks. Bioinformatics, 25(8), 1091–1093. Available at 10.1093/bioinformatics/btp101 19237447 PMC2666812

[pce14420-bib-0012] Birkenbihl, R.P. , Diezel, C. & Somssich, I.E. (2012) Arabidopsis WRKY33 is a key transcriptional regulator of hormonal and metabolic responses toward *Botrytis cinerea* infection. Plant Physiology, 159(1), 266–285. Available at 10.1104/pp.111.192641 22392279 PMC3375964

[pce14420-bib-0013] Bütehorn, B. , Rhody, D. & Franken, P. (2000) Isolation and characterisation of Pitef1 encoding the translation elongation factor EF‐1α of the root endophyte piriformospora indica. Plant Biology, 2(6), 687–692. Available at 10.1055/s-2000-16647

[pce14420-bib-0014] Chen, L.‐Q. , Hou, B.‐H. , Lalonde, S. , Takanaga, H. , Hartung, M.L. , Qu, X.‐Q. et al. (2010) Sugar transporters for intercellular exchange and nutrition of pathogens. Nature, 468(7323), 527–532. Available at 10.1038/nature09606 21107422 PMC3000469

[pce14420-bib-0015] Chen, L.‐Q. , Qu, X.‐Q. , Hou, B.‐H. , Sosso, D. , Osorio, S. , Fernie, A.R. et al. (2012) Sucrose efflux mediated by SWEET proteins as a key step for phloem transport. Science, 335(6065), 207–211. Available at 10.1126/science.1213351 22157085

[pce14420-bib-0016] Conchillo, L.B. , Haro, R. & Benito, B. (2021) K^+^ nutrition exchange in Serendipita‐Arabidopsis symbiosis: study of the fungal K^+^ transporters involved. Front. Ecol. Evol. 9, 789371. Available at 10.3389/fevo.2021.789371

[pce14420-bib-0017] de Bary, A. (1879) Die Erscheinung der Symbiose, Vortrag auf der Versammlung der Naturforscher und Artze zu Cassel. Verlag von K. J. Trübner, pp. 1–30.

[pce14420-bib-0018] Ding, Z.J. , Yan, J.Y. , Li, C.X. , Li, G.X. , Wu, Y.R. & Zheng, S.J. (2015) Transcription factor WRKY46 modulates the development of Arabidopsis lateral roots in osmotic/salt stress conditions via regulation of ABA signaling and auxin homeostasis. The Plant Journal, 84(1), 56–69. Available at 10.1111/tpj.12958 26252246

[pce14420-bib-0019] Dixon, D.P. , Lapthorn, A. & Edwards, R. (2002) Plant glutathione transferases. Genome Biology, 3(3), ReviewS3004. Available at 10.1186/gb-2002-3-3-reviews3004 11897031 PMC139027

[pce14420-bib-0020] Doncheva, N.T. , Morris, J.H. , Gorodkin, J. & Jensen, L.J. (2019) Cytoscape StringApp: network analysis and visualization of proteomics data. Journal of Proteome Research, 18(2), 623–632. Available at 10.1021/acs.jproteome.8b00702 30450911 PMC6800166

[pce14420-bib-0021] Drechsler, N. , Zheng, Y. , Bohner, A. , Nobmann, B. , von Wirén, N. , Kunze, R. et al. (2015) Nitrate‐dependent control of shoot K homeostasis by the nitrate transporter1/peptide transporter family member NPF7.3/NRT1.5 and the stelar K^+^ outward rectifier SKOR in Arabidopsis. Plant Physiology, 169(4), 2832–2847. Available at 10.1104/pp.15.01152 26508776 PMC4677904

[pce14420-bib-0022] Dreyer, I. (2021) Nutrient cycling is an important mechanism for homeostasis in plant cells. Plant Physiology, 187(4), 2246–2261. Available at 10.1093/plphys/kiab217 34890457 PMC8644529

[pce14420-bib-0023] Dreyer, I. & Blatt, M.R. (2009) What makes a gate? The ins and outs of Kv‐like K+ channels in plants. Trends in Plant Science, 14(7), 383–390. Available at 10.1016/j.tplants.2009.04.001 19540150

[pce14420-bib-0024] Dreyer, I. , Porée, F. , Schneider, A. , Mittelstädt, J. , Bertl, A. , Sentenac, H. et al. (2004) Assembly of plant *Shaker*‐like K_out_ channels requires two distinct sites of the channel a‐subunit. Biophysical Journal, 87(2), 858–872. Available at 10.1529/biophysj.103.037671 15298894 PMC1304495

[pce14420-bib-0025] Frerigmann, H. & Gigolashvili, T. (2014) MYB34, MYB51, and MYB122 distinctly regulate indolic glucosinolate biosynthesis in *Arabidopsis thaliana* . Molecular Plant, 7(5), 814–828. Available at 10.1093/mp/ssu004 24431192

[pce14420-bib-0026] Frerigmann, H. , Piślewska‐Bednarek, M. , Sánchez‐Vallet, A. , Molina, A. , Glawischnig, E. , Gigolashvili, T. et al. (2016) Regulation of pathogen‐triggered tryptophan metabolism in *Arabidopsis thaliana* by MYB transcription factors and indole glucosinolate conversion products. Molecular Plant, 9(5), 682–695. Available at 10.1016/j.molp.2016.01.006 26802248

[pce14420-bib-0027] Galkovskyi, T. , Mileyko, Y. , Bucksch, A. , Moore, B. , Symonova, O. , Price, C.A. et al. (2012) GiA roots: software for the high throughput analysis of plant root system architecture. BMC Plant Biology, 12(1), 116. Available at 10.1186/1471-2229-12-116 22834569 PMC3444351

[pce14420-bib-0028] Gaymard, F. , Pilot, G. , Lacombe, B. , Bouchez, D. , Bruneau, D. , Boucherez, J. et al. (1998) Identification and disruption of a plant shaker‐like outward channel involved in K^+^ release into the xylem sap. Cell, 94(5), 647–655. Available at 10.1016/S0092-8674(00)81606-2 9741629

[pce14420-bib-0029] Gierth, M. , Maser, P. & Schroeder, J.I. (2005) The potassium transporter AtHAK5 functions in K^+^ deprivation‐induced high‐affinity K^+^ uptake and AKT1 K^+^ channel contribution to K^+^ uptake kinetics in Arabidopsis roots. Plant Physiology, 137(3), 1105–1114. Available at 10.1104/pp.104.057216 15734909 PMC1065410

[pce14420-bib-0030] Glawischnig, E. (2007) Camalexin. Phytochemistry, 68(4), 401–406. Available at 10.1016/j.phytochem.2006.12.005 17217970

[pce14420-bib-0031] González‐Teuber, M. , Urzúa, A. , Plaza, P. & Bascuñán‐Godoy, L. (2018) Effects of root endophytic fungi on response of *Chenopodium quinoa* to drought stress. Plant Ecology, 219(3), 231–240. Available at 10.1007/s11258-017-0791-1

[pce14420-bib-0032] Gullner, G. , Komives, T. , Király, L. & Schröder, P. (2018) Glutathione S‐transferase enzymes in plant‐pathogen interactions. Frontiers in Plant Science, 9(1836), 1836. Available at 10.3389/fpls.2018.01836 30622544 PMC6308375

[pce14420-bib-0033] Herger, A. , Dünser, K. , Kleine‐Vehn, J. & Ringli, C. (2019) Leucine‐rich repeat extensin proteins and their role in cell wall sensing. Current Biology, 29(17), R851–R858. Available at 10.1016/j.cub.2019.07.039 31505187

[pce14420-bib-0034] Hertig, M. , Taliaferro, W.H. & Schwartz, B. (1937) The terms symbiosis, symbiont and symbiote. Journal of Parasitology, 23, 326–329.

[pce14420-bib-0035] Jacobs, S. , Zechmann, B. , Molitor, A. , Trujillo, M. , Petutschnig, E. , Lipka, V. et al. (2011) Broad‐spectrum suppression of innate immunity is required for colonization of Arabidopsis roots by the fungus *Piriformospora indica* . Plant Physiology, 156(2), 726–740. Available at 10.1104/pp.111.176446 21474434 PMC3177271

[pce14420-bib-0036] Jogawat, A. , Meena, M.K. , Kundu, A. , Varma, M. & Vadassery, J. (2020) Calcium channel CNGC19 mediates basal defense signaling to regulate colonization by *Piriformospora indica* in Arabidopsis roots. Journal of Experimental Botany, 71(9), 2752–2768. Available at 10.1093/jxb/eraa028 31957790 PMC7210775

[pce14420-bib-0037] Jogawat, A. , Vadassery, J. , Verma, N. , Oelmüller, R. , Dua, M. , Nevo, E. et al. (2016) PiHOG1, a stress regulator MAP kinase from the root endophyte fungus *Piriformospora indica*, confers salinity stress tolerance in rice plants. Scientific Reports, 6, 36765. Available at 10.1038/srep36765 27849025 PMC5111105

[pce14420-bib-0038] Johnson, J.M. , Sherameti, I. , Nongbri, P.L. & Oelmüller, R. (2013) Standardized conditions to study beneficial and nonbeneficial traits in the *Piriformospora indica*/*Arabidopsis thaliana* interaction. In: Varma, A. , Kost, G. & Oelmüller, R. (Eds.) Piriformospora indica: Sebacinales and their biotechnological applications. Springer Berlin Heidelberg, pp. 325–343.

[pce14420-bib-0039] Jost, R. , Berkowitz, O. & Masle, J. (2007) Magnetic quantitative reverse transcription PCR: a high‐throughput method for mRNA extraction and quantitative reverse transcription PCR. Biotechniques, 43(2), 206–211. Available at 10.2144/000112534 17824388

[pce14420-bib-0040] Kiba, T. & Krapp, A. (2016) Plant nitrogen acquisition under low availability: regulation of uptake and root architecture. Plant and Cell Physiology, 57(4), 707–714. Available at 10.1093/pcp/pcw052 27025887 PMC4836452

[pce14420-bib-0041] Kim, D. , Paggi, J.M. , Park, C. , Bennett, C. & Salzberg, S.L. (2019) Graph‐based genome alignment and genotyping with HISAT2 and HISAT‐genotype. Nature Biotechnology, 37(8), 907–915. Available at 10.1038/s41587-019-0201-4 PMC7605509 31375807

[pce14420-bib-0042] Konishi, M. & Yanagisawa, S. (2013) Arabidopsis NIN‐like transcription factors have a central role in nitrate signalling. Nature Communications, 4(1), 1617. Available at 10.1038/ncomms2621 23511481

[pce14420-bib-0043] Krouk, G. & Kiba, T. (2020) Nitrogen and phosphorus interactions in plants: from agronomic to physiological and molecular insights. Current Opinion in Plant Biology, 57, 104–109. Available at 10.1016/j.pbi.2020.07.002 32882570

[pce14420-bib-0044] Kudla, J. , Batistič, O. & Hashimoto, K. (2010) Calcium signals: the lead currency of plant information processing. The Plant Cell, 22(3), 541–563. Available at 10.1105/tpc.109.072686 20354197 PMC2861448

[pce14420-bib-0045] Kudla, J. , Becker, D. , Grill, E. , Hedrich, R. , Hippler, M. , Kummer, U. et al. (2018) Advances and current challenges in calcium signaling. New Phytologist, 218(2), 414–431. Available at 10.1111/nph.14966 29332310

[pce14420-bib-0046] Lahrmann, U. , Strehmel, N. , Langen, G. , Frerigmann, H. , Leson, L. , Ding, Y. et al. (2015) Mutualistic root endophytism is not associated with the reduction of saprotrophic traits and requires a noncompromised plant innate immunity. New Phytologist, 207(3), 841–857. Available at 10.1111/nph.13411 25919406

[pce14420-bib-0047] Lan, W.‐Z. , Lee, S.‐C. , Che, Y.‐F. , Jiang, Y.‐Q. & Luan, S. (2011) Mechanistic analysis of AKT1 regulation by the CBL–CIPK–PP2CA interactions. Molecular Plant, 4(3), 527–536. Available at 10.1093/mp/ssr031 21596690

[pce14420-bib-0048] Lee, S.C. , Lan, W.‐Z. , Kim, B.‐G. , Li, L. , Cheong, Y.H. , Pandey, G.K. et al. (2007) A protein phosphorylation/dephosphorylation network regulates a plant potassium channel. Proceedings of the National Academy of Sciences, 104(40), 15959–15964. Available at 10.1073/pnas.0707912104 PMC2000415 17898163

[pce14420-bib-0049] Li, J. , Zhong, R. & Palva, E.T. (2017) WRKY70 and its homolog WRKY54 negatively modulate the cell wall‐associated defenses to necrotrophic pathogens in Arabidopsis. PLoS One, 12(8), e0183731. Available at 10.1371/journal.pone.0183731 28837631 PMC5570282

[pce14420-bib-0050] Liu, K.‐H. & Tsay, Y.‐F. (2003) Switching between the two action modes of the dual‐affinity nitrate transporter CHL1 by phosphorylation. The EMBO Journal, 22(5), 1005–1013. Available at 10.1093/emboj/cdg118 12606566 PMC150351

[pce14420-bib-0051] Livak, K.J. & Schmittgen, T.D. (2001) Analysis of relative gene expression data using real‐time quantitative PCR and the 2(‐delta delta C(T)) method. Methods, 25(4), 402–408. Available at 10.1006/meth.2001.1262 11846609

[pce14420-bib-0052] Lorkovic, Z.J. , Lehner, R. , Forstner, C. & Barta, A. (2005) Evolutionary conservation of minor U12‐type spliceosome between plants and humans. RNA, 11(7), 1095–1107. Available at 10.1261/rna.2440305 15987817 PMC1370794

[pce14420-bib-0053] Love, M.I. , Huber, W. & Anders, S. (2014) Moderated estimation of fold change and dispersion for RNA‐seq data with DESeq. 2. Genome Biology, 15(12), 550. Available at 10.1186/s13059-014-0550-8 25516281 PMC4302049

[pce14420-bib-0054] Ma, Q. , Tang, R.‐J. , Zheng, X.‐J. , Wang, S.‐M. & Luan, S. (2015) The calcium sensor CBL7 modulates plant responses to low nitrate in *Arabidopsis* . Biochemical and Biophysical Research Communications, 468(1), 59–65. Available at 10.1016/j.bbrc.2015.10.164 26549233

[pce14420-bib-0055] Maierhofer, T. , Diekmann, M. , Offenborn, J.N. , Lind, C. , Bauer, H. , Hashimoto, K. et al. (2014) Site‐ and kinase‐specific phosphorylation‐mediated activation of SLAC1, a guard cell anion channel stimulated by abscisic acid. Science Signaling, 7(342), ra86. Available at 10.1126/scisignal.2005703 25205850

[pce14420-bib-0056] Malka, S.K. & Cheng, Y. (2017) Possible interactions between the biosynthetic pathways of indole glucosinolate and auxin. Frontiers in Plant Science, 8(2131), 2131. Available at 10.3389/fpls.2017.02131 29312389 PMC5735125

[pce14420-bib-0057] Marchive, C. , Roudier, F. , Castaings, L. , Bréhaut, V. , Blondet, E. , Colot, V. et al. (2013) Nuclear retention of the transcription factor NLP7 orchestrates the early response to nitrate in plants. Nature Communications, 4, 1713. Available at 10.1038/ncomms2650 23591880

[pce14420-bib-0058] Mensah, R.A. , Li, D. , Liu, F. , Tian, N. , Sun, X. , Hao, X. et al. (2020) Versatile *Piriformospora indica* and its potential applications in horticultural crops. Horticultural Plant Journal, 6(2), 111–121. Available at 10.1016/j.hpj.2020.01.002

[pce14420-bib-0059] Merico, D. , Isserlin, R. , Stueker, O. , Emili, A. & Bader, G.D. (2010) Enrichment map: a network‐based method for gene set enrichment visualization and interpretation. PLoS One, 5(11), e13984. Available at 10.1371/journal.pone.0013984 21085593 PMC2981572

[pce14420-bib-0060] Millet, Y.A. , Danna, C.H. , Clay, N.K. , Songnuan, W. , Simon, M.D. , Werck‐Reichhart, D. et al. (2010) Innate immune responses activated in Arabidopsis roots by microbe‐associated molecular patterns. The Plant Cell, 22(3), 973–990. Available at 10.1105/tpc.109.069658 20348432 PMC2861455

[pce14420-bib-0061] Monaghan, J. , Xu, F. , Xu, S. , Zhang, Y. & Li, X. (2010) Two putative RNA‐binding proteins function with unequal genetic redundancy in the MOS4‐associated complex. Plant Physiology, 154(4), 1783–1793. Available at 10.1104/pp.110.158931 20943852 PMC2996007

[pce14420-bib-0062] Murashige, T. & Skoog, F. (1962) A revised medium for rapid growth and bio assays with tobacco tissue cultures. Physiologia Plantarum, 15(3), 473–497. Available at 10.1111/j.1399-3054.1962.tb08052.x

[pce14420-bib-0063] Nolte, H. , MacVicar, T.D. , Tellkamp, F. & Krüger, M. (2018) Instant clue: a software suite for interactive data visualization and analysis. Scientific Reports, 8(1), 12648. Available at 10.1038/s41598-018-31154-6 30140043 PMC6107636

[pce14420-bib-0064] Nongbri, P.L. , Johnson, J.M. , Sherameti, I. , Glawischnig, E. , Halkier, B.A. & Oelmuller, R. (2012) Indole‐3‐acetaldoxime‐derived compounds restrict root colonization in the beneficial interaction between Arabidopsis roots and the endophyte *Piriformospora indica* . Molecular Plant‐Microbe Interactions, 25(9), 1186–1197. Available at 10.1094/MPMI-03-12-0071-R 22852809

[pce14420-bib-0065] Ohta, M. , Guo, Y. , Halfter, U. & Zhu, J.K. (2003) A novel domain in the protein kinase SOS2 mediates interaction with the protein phosphatase 2C ABI2. Proceedings of the National Academy of Sciences of the United States of America, 100(20), 11771–11776. Available at 10.1073/pnas.2034853100 14504388 PMC208833

[pce14420-bib-0066] Oñate‐Sánchez, L. & Vicente‐Carbajosa, J. (2008) DNA‐free RNA isolation protocols for *Arabidopsis thaliana*, including seeds and siliques. BMC Research Notes, 1, 93. Available at 10.1186/1756-0500-1-93 18937828 PMC2613888

[pce14420-bib-0067] Pérez‐Alonso, M.M. , Ortiz‐García, P. , Moya‐Cuevas, J. , Lehmann, T. , Sánchez‐Parra, B. , Björk, R.G. et al. (2021) Endogenous indole‐3‐acetamide levels contribute to the crosstalk between auxin and abscisic acid, and trigger plant stress responses in *Arabidopsis thaliana* . Journal of Experimental Botany, 72(2), 459–475. Available at 10.1093/jxb/eraa485 33068437 PMC7853601

[pce14420-bib-0068] Peskan‐Berghofer, T. , Shahollari, B. , Giong, P.H. , Hehl, S. , Markert, C. , Blanke, V. et al. (2004) Association of *Piriformospora indica* with *Arabidopsis thaliana* roots represents a novel system to study beneficial plant–microbe interactions and involves early plant protein modifications in the endoplasmic reticulum and at the plasma membrane. Physiologia Plantarum, 122(4), 465–477. Available at 10.1111/j.1399-3054.2004.00424.x

[pce14420-bib-0069] Pfister, A. , Barberon, M. , Alassimone, J. , Kalmbach, L. , Lee, Y. , Vermeer, J.E. et al. (2014) A receptor‐like kinase mutant with absent endodermal diffusion barrier displays selective nutrient homeostasis defects. eLife, 3, e03115. Available at 10.7554/eLife.03115 25233277 PMC4164916

[pce14420-bib-0070] Pivato, M. & Ballottari, M. (2021) *Chlamydomonas reinhardtii* cellular compartments and their contribution to intracellular calcium signalling. Journal of Experimental Botany, 72(15), 5312–5335. Available at 10.1093/jxb/erab212 34077536 PMC8318260

[pce14420-bib-0071] Prasad, D. , Verma, N. , Bakshi, M. , Narayan, O.P. , Singh, A.K. , Dua, M. et al. (2018) Functional characterization of a magnesium transporter of root endophytic fungus *Piriformospora indica* . Frontiers in Microbiology, 9, 3231. Available at 10.3389/fmicb.2018.03231 30687249 PMC6333687

[pce14420-bib-0072] Ragel, P. , Ródenas, R. , García‐Martín, E. , Andrés, Z. , Villalta, I. , Nieves‐Cordones, M. et al. (2015) The CBL‐interacting protein kinase CIPK23 regulates HAK5‐mediated high‐affinity K+ uptake in Arabidopsis roots. Plant Physiology, 169(4), 2863–2873. Available at 10.1104/pp.15.01401 26474642 PMC4677917

[pce14420-bib-0073] Rodriguez, R.J. , Redman, R.S. & Henson, J.M. (2004) The role of fungal symbioses in the adaptation of plants to high stress environments. Mitigation and Adaptation Strategies for Global Change, 9(3), 261–272. Available at 10.1023/b:Miti.0000029922.31110.97

[pce14420-bib-0074] Rodríguez‐Navarro, A. & Ramos, J. (1984) Dual system for potassium transport in *Saccharomyces cerevisiae* . Journal of Bacteriology, 159(3), 940–945. Available at 10.1128/jb.159.3.940-945.1984 6384187 PMC215750

[pce14420-bib-0075] Saga, H. , Ogawa, T. , Kai, K. , Suzuki, H. , Ogata, Y. , Sakurai, N. et al. (2012) Identification and characterization of ANAC042, a transcription factor family gene involved in the regulation of camalexin biosynthesis in arabidopsis. Molecular Plant‐Microbe Interactions®, 25(5), 684–696. Available at 10.1094/mpmi-09-11-0244 22295908

[pce14420-bib-0076] Schliebner, I. , Pribil, M. , Zuhlke, J. , Dietzmann, A. & Leister, D. (2008) A survey of chloroplast protein kinases and phosphatases in *Arabidopsis thaliana* . Current Genomics, 9(3), 184–190. Available at 10.2174/138920208784340740 19440515 PMC2679645

[pce14420-bib-0077] Shannon, P. , Markiel, A. , Ozier, O. , Baliga, N.S. , Wang, J.T. , Ramage, D. et al. (2003) Cytoscape: a software environment for integrated models of biomolecular interaction networks. Genome Research, 13(11), 2498–2504. Available at 10.1101/gr.1239303 14597658 PMC403769

[pce14420-bib-0078] Shin, R. & Schachtman, D.P. (2004) Hydrogen peroxide mediates plant root cell response to nutrient deprivation. Proceedings of the National Academy of Sciences of the United States of America, 101(23), 8827–8832. Available at 10.1073/pnas.0401707101 15173595 PMC423280

[pce14420-bib-0079] Sivitz, A.B. , Hermand, V. , Curie, C. & Vert, G. (2012) Arabidopsis bHLH100 and bHLH101 control iron homeostasis via a FIT‐independent pathway. PLoS One, 7(9), e44843. Available at 10.1371/journal.pone.0044843 22984573 PMC3439455

[pce14420-bib-0080] Sun, C. , Shao, Y. , Vahabi, K. , Lu, J. , Bhattacharya, S. , Dong, S. et al. (2014) The beneficial fungus *Piriformospora indica* protects Arabidopsis from *Verticillium dahliae* infection by downregulation plant defense responses. BMC Plant Biology, 14, 268. Available at 10.1186/s12870-014-0268-5 25297988 PMC4198706

[pce14420-bib-0081] Tang, R.‐J. , Wang, C. , Li, K. & Luan, S. (2020) The CBL–CIPK calcium signaling network: unified paradigm from 20 years of discoveries. Trends in Plant Science, 25(6), 604–617. Available at 10.1016/j.tplants.2020.01.009 32407699

[pce14420-bib-0082] Taylor‐Teeples, M. , Lin, L. , de Lucas, M. , Turco, G. , Toal, T.W. , Gaudinier, A. et al. (2015) An Arabidopsis gene regulatory network for secondary cell wall synthesis. Nature, 517(7536), 571–575. Available at 10.1038/nature14099 25533953 PMC4333722

[pce14420-bib-0083] Thimm, O. , Bläsing, O. , Gibon, Y. , Nagel, A. , Meyer, S. , Krüger, P. et al. (2004) MAPMAN: a user‐driven tool to display genomics data sets onto diagrams of metabolic pathways and other biological processes. Plant Journal, 37(6), 914–939. Available at 10.1111/j.1365-313X.2004.02016.x 14996223

[pce14420-bib-0084] Thor, K. (2019) Calcium—nutrient and messenger. Frontiers in Plant Science, 10(440), 440. Available at 10.3389/fpls.2019.00440 31073302 PMC6495005

[pce14420-bib-0085] Tong, T. , Li, Q. , Jiang, W. , Chen, G. , Xue, D. , Deng, F. et al. (2021) Molecular evolution of calcium signaling and transport in plant adaptation to abiotic stress. International Journal of Molecular Sciences, 22(22), 12308. 34830190 10.3390/ijms222212308 PMC8618852

[pce14420-bib-0086] Vadassery, J. & Oelmüller, R. (2009) Calcium signaling in pathogenic and beneficial plant microbe interactions: what can we learn from the interaction between *Piriformospora indica* and *Arabidopsis thaliana* . Plant Signaling & Behavior, 4(11), 1024–1027. Available at 10.4161/psb.4.11.9800 19829075 PMC2819509

[pce14420-bib-0087] Vadassery, J. , Ranf, S. , Drzewiecki, C. , Mithöfer, A. , Mazars, C. , Scheel, D. et al. (2009) A cell wall extract from the endophytic fungus *Piriformospora indica* promotes growth of Arabidopsis seedlings and induces intracellular calcium elevation in roots. The Plant Journal, 59(2), 193–206. Available at 10.1111/j.1365-313X.2009.03867.x 19392691

[pce14420-bib-0088] Varma, A. , Savita, V. , Sudha, . , Sahay, N. , Butehorn, B. & Franken, P. (1999) *Piriformospora indica*, a cultivable plant‐growth‐promoting root endophyte. Applied and Environmental Microbiology, 65(6), 2741–2744. Available at 10.1128/AEM.65.6.2741-2744.1999 10347070 PMC91405

[pce14420-bib-0089] Verma, S. , Varma, A. , Rexer, K.‐H. , Hassel, A. , Kost, G. , Sarbhoy, A. et al. (1998) *Piriformospora indica*, gen. et sp. nov., a new root‐colonizing fungus. Mycologia, 90(5), 896–903. Available at 10.1080/00275514.1998.12026983

[pce14420-bib-0090] Waller, F. , Achatz, B. , Baltruschat, H. , Fodor, J. , Becker, K. , Fischer, M. et al. (2005) The endophytic fungus *Piriformospora indica* reprograms barley to salt‐stress tolerance, disease resistance, and higher yield. Proceedings of the National Academy of Sciences of the United States of America, 102(38), 13386–13391. Available at 10.1073/pnas.0504423102 16174735 PMC1224632

[pce14420-bib-0091] Weiss, M. , Waller, F. , Zuccaro, A. & Selosse, M.A. (2016) Sebacinales—one thousand and one interactions with land plants. New Phytologist, 211(1), 20–40. Available at 10.1111/nph.13977 27193559

[pce14420-bib-0092] Wittstock, U. & Burow, M. (2010) Glucosinolate breakdown in Arabidopsis: mechanism, regulation and biological significance. The Arabidopsis Book, 8, e0134. Available at 10.1199/tab.0134 22303260 PMC3244901

[pce14420-bib-0093] Xu, L. , Wu, C. , Oelmüller, R. & Zhang, W. (2018) Role of phytohormones in *Piriformospora indica*‐induced growth promotion and stress tolerance in plants: more questions than answers. Frontiers in Microbiology, 9, 1646. Available at 10.3389/fmicb.2018.01646 30140257 PMC6094092

[pce14420-bib-0094] Yang, Y. , Wu, Y. , Ma, L. , Yang, Z. , Dong, Q. , Li, Q. et al. (2019) The Ca^2+^ sensor SCaBP3/CBL7 modulates plasma membrane H^+^‐ATPase activity and promotes alkali tolerance in arabidopsis. The Plant Cell, 31(6), 1367–1384. Available at 10.1105/tpc.18.00568 30962395 PMC6588306

[pce14420-bib-0095] Zamioudis, C. , Korteland, J. , Van Pelt, J.A. , van Hamersveld, M. , Dombrowski, N. , Bai, Y. et al. (2015) Rhizobacterial volatiles and photosynthesis‐related signals coordinate MYB72 expression in Arabidopsis roots during onset of induced systemic resistance and iron‐deficiency responses. The Plant Journal, 84(2), 309–322. Available at 10.1111/tpj.12995 26307542 PMC5019235

[pce14420-bib-0096] Zhou, J. , Wang, X. , He, Y. , Sang, T. , Wang, P. , Dai, S. et al. (2020) Differential phosphorylation of the transcription factor WRKY33 by the protein kinases CPK5/CPK6 and MPK3/MPK6 cooperatively regulates camalexin biosynthesis in Arabidopsis. The Plant Cell, 32(8), 2621–2638. Available at 10.1105/tpc.19.00971 32439826 PMC7401014

