# Supplementary figures and images for: The calcium sensor CBL7 is required for Serendipita indica‐induced growth stimulation in Arabidopsis thaliana, controlling defense against the endophyte and K+ homoeostasis in the symbiosis
Source: Plant Cell Environ. 2022 Aug 29;45(11):3367–82. doi: 10.1111/pce.14420 (PMC9804297; doi:10.1111/pce.14420)

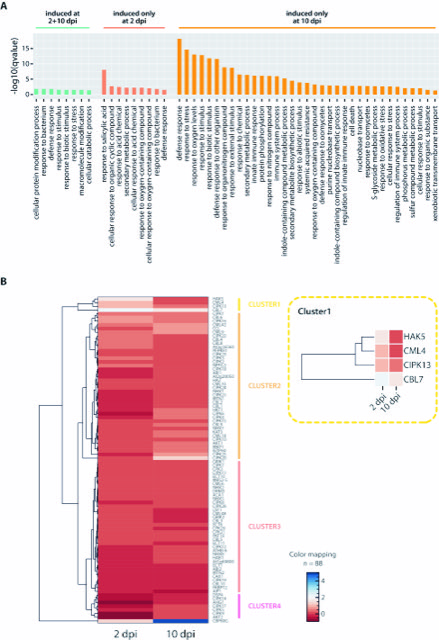

Supplement: Supplementary file 4 — Supporting information. [file PCE-45-3367-s002.jpg]

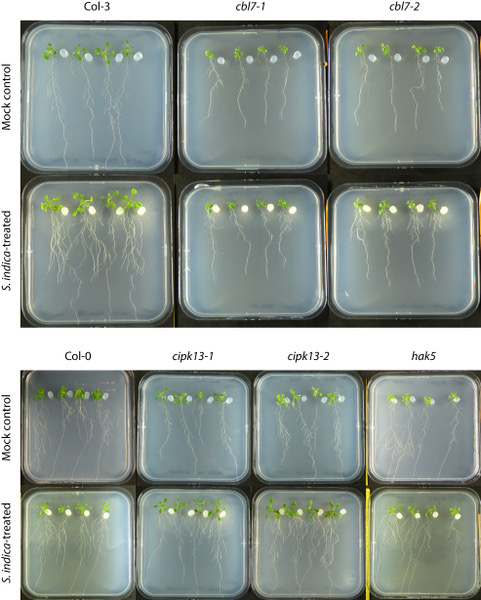

Supplement: Supplementary file 5 — Supporting information. [file PCE-45-3367-s004.jpg]
