# Supplementary material for: The calcium sensor CBL7 is required for Serendipita indica‐induced growth stimulation in Arabidopsis thaliana, controlling defense against the endophyte and K+ homoeostasis in the symbiosis
Source: Plant Cell Environ. 2022 Aug 29;45(11):3367–82. doi: 10.1111/pce.14420 (PMC9804297; doi:10.1111/pce.14420)
Supplement: Supplementary file 6 — Supporting information. [file PCE-45-3367-s003.docx]

# Short legends for Supporting Information

Supplemental Data Sheet 1: Primers used for genotyping, cloning and expression

analysis

Supplemental Data Sheet 2: RNA-seq analysis of *S. indica* and mock treated Arabidopsis wild-type seedlings.

Supplementary Data Sheet 3: RNA-seq analysis of *S. indica* and mock treated Arabidopsis *cbl7-2* and Col-3 seedlings

Supplemental Image 1: GO and cluster analysis of induced DEGs in *S. indica* challenged wt plants compared to mock treated control plants.

Supplemental Image 2: Representative images of mock and *S. indica*-treated Arabidopsis genotypes.
